# Neuronal tracing of oral nerves in a velvet worm—Implications for the evolution of the ecdysozoan brain

**DOI:** 10.3389/fnana.2014.00007

**Published:** 2014-02-26

**Authors:** Christine Martin, Georg Mayer

**Affiliations:** Animal Evolution & Development, Institute of Biology, University of LeipzigLeipzig, Germany

**Keywords:** Onychophora, Ecdysozoa, Cycloneuralia, central nervous system, lip papillae, mouth, arthropods

## Abstract

As one of the closest relatives of arthropods, Onychophora plays an important role in understanding the evolution of arthropod body plans. Currently there is controversy surrounding the evolution of the brain among the ecdysozoan clades, which shows a collar-shaped, circumoral organization in cycloneuralians but a ganglionic architecture in panarthropods. Based on the innervation pattern of lip papillae surrounding the mouth, the onychophoran brain has been interpreted as a circumoral ring, suggesting that this organization is an ancestral feature of Ecdysozoa. However, this interpretation is inconsistent with other published data. To explore the evolutionary origin of the onychophoran mouth and to shed light on the evolution of the ecdysozoan brains, we analyzed the innervation pattern and morphogenesis of the oral lip papillae in the onychophoran *Euperipatoides rowelli* using DNA labeling, immunocytochemistry, and neuronal tracing techniques. Our morphogenetic data revealed that the seven paired and one unpaired oral lip papillae arise from three anterior-most body segments. Retrograde fills show that only the first and the third nerves supplying the lip papillae are associated with cell bodies within the brain, whereas the second nerve exclusively receives fibers from somata of peripheral neurons located in the lip papillae. According to our anterograde fills and immunocytochemical data, the first nerve supplies the anterior-most pair of lip papillae, whereas the second and the third nerves are associated with the second to fifth and second to eighth lip papillae, respectively. These data suggest that the lip papillae of *E. rowelli* are mainly innervated by the proto- and deutocerebrum, whereas there are only a few additional cell bodies situated posterior to the brain. According to these findings, the overall innervation pattern of the oral lip papillae in *E. rowelli* is incompatible with the interpretation of the onychophoran brain as a modified circumoral ring.

## Introduction

**Figure 1:**
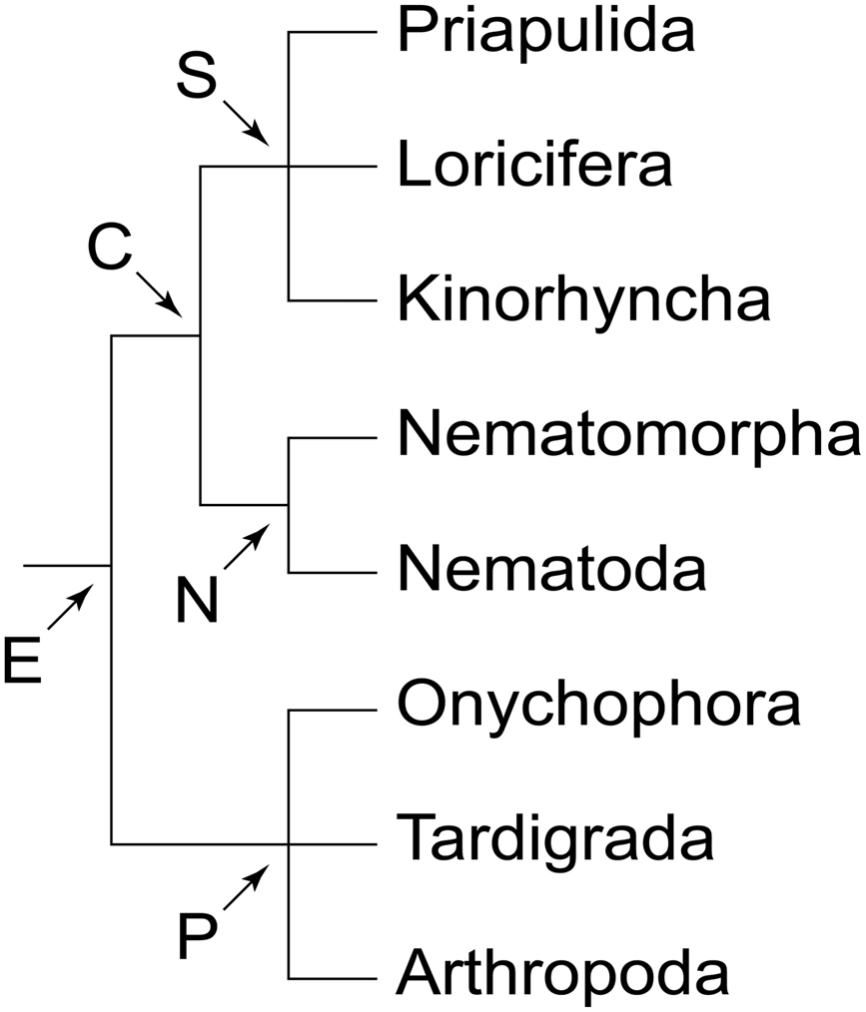
**Phylogeny of Ecdysozoa (molting animals)**. Trichotomies indicate unresolved relationships. Phylogenetic relationships combined after Mayer and Whitington (2009b) and Nielsen (2012). Abbreviations: C, Cycloneuralia; E, Ecdysozoa; N, Nematoida; P, Panarthropoda; S, Scalidophora.

There are two major types of brain among ecdysozoans or molting animals (Figure 1). Representatives of priapulids, kinorhynchs, nematodes and allies possess a circumoral, collar-shaped brain, with anterior and posterior rings of perikarya separated by a ring-like neuropil, hence the name Cycloneuralia for the clade including these taxa (Bullock and Horridge, 1965; Ahlrichs, 1995; Nielsen, 2012). In contrast, members of the second major clade of Ecdysozoa, the Panarthropoda (Onychophora + Tardigrada + Arthropoda), exhibit a typical ganglionic, bilaterally symmetric brain situated dorsally within the head (e.g., Homberg, 1991; Strausfeld et al., 2006a; Mayer et al., 2010, 2013a,b). Thus, the question arises of whether a cycloneuralian-like or a panarthropod-like brain was present in the last common ancestor of Ecdysozoa.

**Figure 2:**
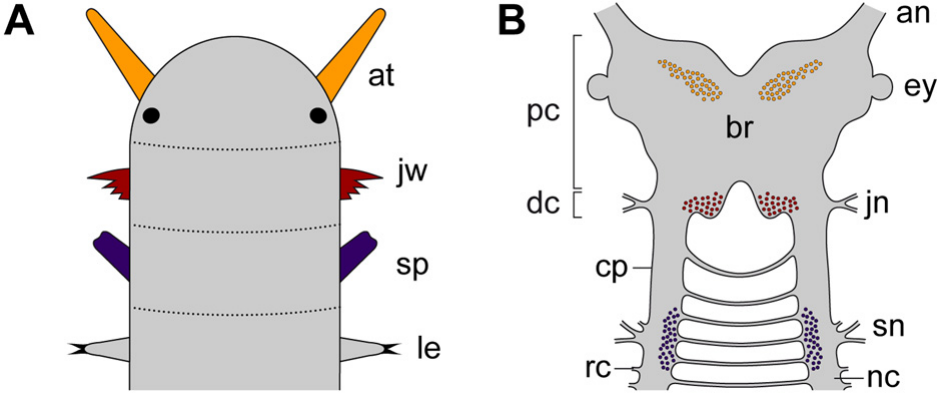
**Diagrams illustrating segmental identity of modified cephalic appendages and position of corresponding neuronal somata in the central nervous system of Onychophora**. Modified from Mayer et al. (2010). **(A)** Assignment of four anterior-most pairs of limbs to corresponding segments (demarcated by dotted lines). Black-filled circles indicate the position of eyes. **(B)** Position of neuronal cell bodies innervating the three pairs of cephalic appendages, including the antennae, the jaws, and the slime papillae. Note that the somata of neurons supplying the slime papillae lie outside the brain. Abbreviations: an, antennal nerve; at, antenna; br, brain; cp, connecting piece; dc; deutocerebral region; ey, eye; jn, jaw nerve; jw, jaw; le, first walking leg; nc, nerve cord; pc, protocerebral region; rc, ring commissure; sn, slime papilla nerve; sp; slime papilla.

As one of the closest relatives of arthropods, Onychophora (velvet worms) occupies a key position for clarifying this issue (Whitington and Mayer, 2011). Although the onychophoran head comprises three segments, each with a pair of modified appendages (antennae, jaws and slime papillae), localization of the neuronal somata supplying these appendages has demonstrated that the region innervating the slime papillae is not differentiated as part of the brain, but rather belongs to the nerve cord (Figures 2A,B). This suggests that onychophorans have a bipartite brain, which consists of the proto- and deutocerebrum, whereas the tritocerebrum evolved in the arthropod lineage (Mayer et al., 2010).

Like in arthropods, the protocerebrum of onychophorans is associated with the eyes and contains the central body and the mushroom bodies (Holmgren, 1916; Hanström, 1928; Schürmann, 1987a; Strausfeld et al., 2006a,b; Mayer et al., 2010). In contrast to arthropods, however, it also innervates a pair of antennae, which are not homologous to the (first) antennae of myriapods, crustaceans and hexapods, as these are supplied by the deutocerebrum (Scholtz and Edgecombe, 2006; Strausfeld et al., 2006a,b). The deutocerebral region of the onychophoran brain is instead associated with the appendages of the second body segment, i.e., the jaws (Figures 2A,B). This region comprises the posterior-most part of the brain and is followed by the region innervating the slime papillae, which corresponds to the tritocerebrum of arthropods (Mayer et al., 2010). The region associated with the slime papillae nerves is connected to the brain via a pair of cords (referred to herein as “connecting pieces”) that resemble the nerve cords, except that the ring commissures are lacking (Figure 2B). The connecting pieces have been regarded as “circumpharyngeal connectives” in the literature (Eriksson and Budd, 2000), but this term is inappropriate, as in contrast to typical connectives they are accompanied by neuronal somata (Strausfeld et al., 2006a).

While most previous studies have provided no evidence of a circumoral, collar-shaped organization of the onychophoran brain (Holmgren, 1916; Hanström, 1928, 1935; Fedorow, 1929; Henry, 1948a; Schürmann, 1987b; Strausfeld et al., 2006a,b; Mayer et al., 2010), Eriksson and Budd (2000) found some indication for such an organization in the last common ancestor of Onychophora by analyzing the innervation pattern of the mouth in the onychophoran *Euperipatoides kanangrensis*. If confirmed, this finding would suggest that a circumoral rather than a dorsal brain was present in the last common ancestor of Ecdysozoa.

However, there are two major inconsistencies in the interpretation of Eriksson and Budd (2000). First, the authors did not consider the incorporation of the second pair of segmental appendages, i.e., the jaws, into the mouth cavity, which has occurred in the onychophoran lineage and implies an independent origin of the adult mouth (Ou et al., 2012). Second, the bipartite organization of the onychophoran brain (Holmgren, 1916; Hanström, 1928; Mayer et al., 2010) clearly contradicts the assumption that the oral lip papillae of onychophorans are innervated by three segmental brain regions (Eriksson and Budd, 2000). Hence, clarifying the segmental identity of the oral lip papillae as well as the position of associated neurons within the central nervous system might shed light on the evolution of the onychophoran mouth and help to answer the question of whether a cycloneuralian-like or a panarthropod-like brain was present in the last common ancestor of Ecdysozoa.

Despite an increasing number of developmental studies of the onychophoran head (e.g., Eriksson et al., 2003, 2010; Walker and Tait, 2004; Mayer et al., 2005; Ou et al., 2012; Treffkorn and Mayer, 2013), only limited information is available on the segmental origin of lip papillae surrounding the onychophoran mouth opening. While the anterior-most lip papillae most likely arise from the first (antennal) body segment, the segmental origin of the lateral and posterior lip papillae is unclear, as distinct segmental borders are not evident in the onychophoran embryo (Sedgwick, 1885; von Kennel, 1888; Evans, 1901; Walker and Tait, 2004; Mayer et al., 2005; Mayer and Whitington, 2009a; Ou et al., 2012). The study of segmentation genes has not been helpful for clarifying this issue, as the expression domains of these genes do not seem to extend into the lip papillae (Vitzthum et al., 1996; Eriksson et al., 2009; Janssen and Budd, 2013).

The major objective of our study is therefore: (1) to clarify the number and position of lip papillae in adult specimens of the onychophoran *Euperipatoides rowelli*, as these differ among the species (Manton and Heatley, 1937; Oliveira et al., 2012); (2) to analyse the spatial relationship of lip papillae to other cephalic structures throughout development, as this might help to clarify the segmental identity of each lip papilla; and (3) to localize the somata of neurons supplying the lip papillae, as this would reveal a circumoral arrangement of the nervous system, if present (Eriksson and Budd, 2000). These data from the onychophoran *E. rowelli* will provide insights into the origin of the onychophoran mouth and will help clarify the evolutionary changes of brain architecture among the ecdysozoan taxa.

## Materials and methods

### Specimen collection and maintenance

Specimens of *E. rowelli* Reid, 1996 (Onychophora, Peripatopsidae) were collected from rotted logs in the Tallaganda State Forest (New South Wales, Australia; 35°26'S, 149°33'E, 954 m) in January 2013. Permission for specimen collection was obtained from the Forestry Commission of New South Wales (permit no. SPPR0008). The animals were kept in plastic jars (diameter 55 mm, height 70 mm) with perforated lids at 18°C as described previously (Baer and Mayer, 2012) and fed with decapitated crickets every 4 weeks.

### Immunohistochemistry and embryo staging

Females of *E. rowelli* were anesthetized in chloroform vapor for 15–20 s. The embryos were dissected in a physiological saline based on the composition of the onychophoran hemolymph (Robson et al., 1966) and fixed in 4% paraformaldehyde (PFA) in phosphate-buffered saline (PBS; 0.1M, pH 7.4) overnight. The embryos were washed in PBS and preserved in PBS containing 0.05% sodium azide. They were staged according to Walker and Tait (2004) except for stage V embryos, which were classified in a more restrictive way by using the following features: (1) cerebral grooves (anlagen of the hypocerebral organs) appear as longitudinal slits in the middle of each cephalic lobe, and (2) the anlagen of the last (15th) pair of walking legs have occurred.

For immunocytochemistry, whole embryonic heads were used. They were first blocked in 10% Normal-Goat-Serum (Sigma-Aldrich, in PBS-TX) for 2.5 h and then incubated in a solution containing a primary antibody (mouse anti-acetylated α-tubulin; diluted 1:1000 in PBS-TX) for 48 h. After additional washing steps in PBS-TX, the heads were incubated with one of the two secondary antibodies (Alexa Fluor® 488 or Alexa Fluor® 568 goat anti-mouse; Invitrogen, Carlsbad, CA, USA; each diluted 1:500 in PBS-TX) for another 48 h. The specimens were then washed in PBS and the DNA marker Bisbenzimide (H33258, 1 μg/ml in PBS; Sigma-Aldrich) was applied for counter staining. The heads were mounted between two coverslips in Vectashield® Mounting Medium (Vector Laboratories, Burlingame, CA, USA).

For f-actin staining, the fixed embryos were rinsed in several changes of PBS and then incubated for 1 h in a solution containing phalloidin-rhodamine (Molecular Probes, catalog no. R-415300; to the 300 U stock, 1.5 ml methanol was added, and 10 μ l aliquots were stored at −20°C; prior to use, methanol was evaporated and 200 μl PBS was added to each aliquot). After additional rinses in PBS, the DNA-selective fluorescent dye Bisbenzimide was applied for 15 min as described above. After several rinses in PBS, the embryos were either mounted directly on glass slides in Vectashield® Mounting Medium or dehydrated through a methanol or isopropanol series and mounted either on glass slides or between two coverslips in Murray Clear (a 2:1 mixture of benzyl benzoate and benzyl alcohol) as described previously (Mayer and Whitington, 2009a,b).

### Retrograde and anterograde fills of cephalic nerves

For neuronal tracing, adult specimens were anesthetized in chloroform vapor for 20–30 s and cut open longitudinally along the dorsal or ventral side, depending on the selected nerves, using fine scissors. The brains with anterior portions of descending nerve cords and mouth lips with associated nerves were dissected in physiological saline and pinned down with tungsten needles in small Petri dishes coated with Sylgard® (184 Silicone Elastomer Kit, DowCorning GmbH, Wiesbaden, Germany). A well of Vaseline was built around each nerve, after which the saline was removed from each well and replaced with distilled water, to which a few crystals of dextran coupled to either tetramethylrhodamine or fluorescein (MW 3000, lysine-fixable; Molecular Probes, Eugene, USA) were added (Pflüger and Field, 1999). The preparations were then kept in the dark for 12–15 h at 4°C, after which the well containing dextran was carefully removed. After a quick rinse, the preparations were fixed in 4% PFA in PBS for 2 h at 4°C. They were then washed several times in PBS, dehydrated through an ethanol series (50%, 70%, 90%, 95%, 2 × 100%; 10 min each), cleared in methyl salicylate and mounted between two coverslips.

### Confocal microscopy, light microscopy, and image processing

Whole mounts of brains, dissected mouth lips, and embryos were analyzed with a fluorescence microscope (Leica Leitz DMR; Leica Microsystems, Wetzlar, Germany) and a confocal laser-scanning microscope (Leica TCS STED; Leica Microsystems). Confocal image stacks were processed with Leica AS AF v2.3.5 (Leica Microsystems), Zeiss LSM Image Browser Version 4.2.0.121 (Carl Zeiss MicroImaging GmbH, Jena, Germany) and IMARIS 7.2.1 (Bitplane, Zurich, Switzerland). Final panels and diagrams were designed using Adobe (San Jose, California, USA) Photoshop CS5 and Illustrator CS5.

## Results

### Structure and position of the mouth in the onychophoran *Euperipatoides rowelli*

**Figure 3:**
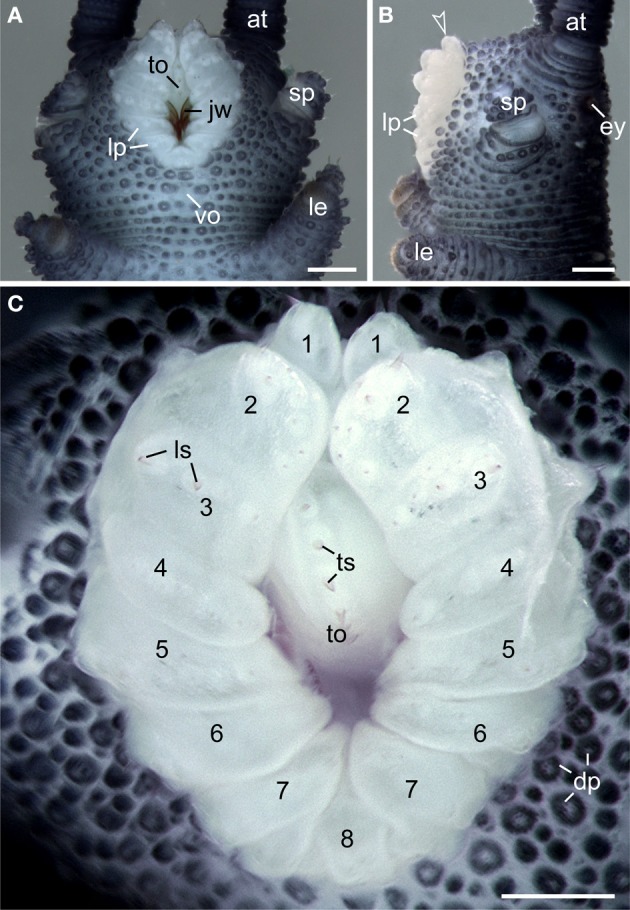
**Structure and position of the adult mouth in the onychophoran *Euperipatoides rowelli***. Stereomicrographs; anterior is up in all images. **(A)** Head in ventral view. **(B)** Head in lateral view. Arrowhead points to the anterior-most pair of lip papillae. **(C)** Detail of lip papillae (numbered). Note that the mouth opening is encompassed by seven paired papillae (no. 1–7) und one unpaired posterior lip papilla (no. 8). Abbreviations: at, antenna; ey, eye; dp, dermal papillae; jw, jaw; le, first walking leg; lp, lip papillae; ls, lip sensilla; sp, slime papilla; to, tongue; ts, tongue sensilla; vo, ventral organ. Scale bars: 250 μm **(A–C)**.

Like in other onychophoran species, the mouth of *E. rowelli* is an oval-shaped cephalic structure, which is located ventrally on the head between the two slime papillae (Figures 3A,B). The mouth cavity contains a pair of sclerotized jaws and an unpaired tongue (Figures 3A,C). The mouth opening is surrounded by a ring of seven paired and one unpaired posterior lip papillae that in contrast to the surrounding integument are unpigmented (Figures 3A–C). The anterior-most lip papillae are the smallest and globular in shape (number 1 in Figure 3C). This pair is followed posteriorly by the largest, nearly quadrangular, second pair (number 2 in Figure 3C) and five pairs of elongated papillae that converge toward the mouth opening (numbered 3–7 in Figure 3C). In contrast, the posterior-most lip papilla lies mid-ventrally and is triangular in shape (number 8 in Figure 3C). Although this lip papilla seems to be unpaired, its paired nature is recognizable by the bilaterally symmetric arrangement of cone-shaped sensilla on its surface (see Supplementary Figure S1), which are also found on all other lip papillae and the tongue (Figure 3C).

### Morphogenesis of lip papillae surrounding the mouth opening in *Euperipatoides rowelli*

**Figure 4:**
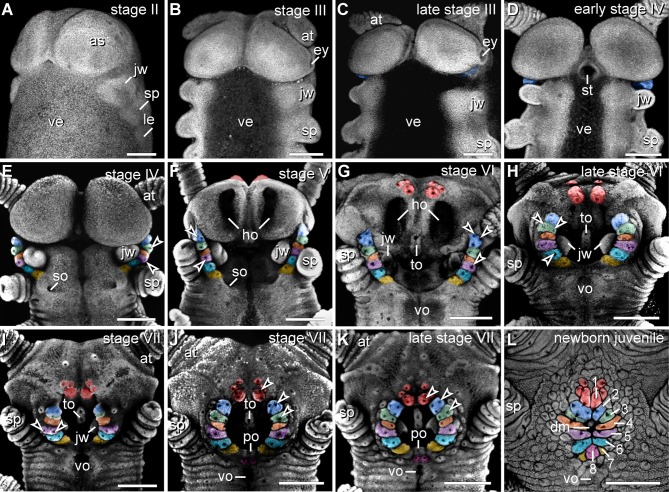
**Morphogenesis of lip papillae encompassing the definitive mouth opening in *Euperipatoides rowelli*. (A–L)** Confocal micrographs of embryonic heads at successive developmental stages in ventrolateral and ventral views. DNA labeling with Bisbenzimide. Developing lip papillae are highlighted by artificial colors. Note that the first pair of lip papillae (number 1 in **L** and red in **F–L**) is associated with the first body segment and that it moves from an anterior to a ventral position during development. Note also that the remaining pairs of lip papillae (numbers 2–7 in **L**) arise lateral to the anlagen of jaws, i.e., in the second body segment, whereas the posterior-most papilla (number 8 in **L**) originates from a paired anlage in the third body segment (magenta in **J–L**). Arrowheads in **(E–K)** point to the developing lip sensilla. Abbreviations: as, anlage of the antennal segment (=cephalic lobe); at, presumptive antenna; dm, definitive mouth opening; ey, eye anlage; ho, anlagen of the hypocerebral organs; jw, developing jaw; le, anlage of the first walking leg; po, embryonic preventral organ of the slime papilla segment; so, openings of the developing salivary glands; sp, developing slime papilla; st, stomodeum; to, embryonic tongue; ve, ventral extraembryonic tissue; vo, developing ventral organ of the slime papilla segment. Scale bars: 250 μm **(A–L)**.

**Figure 5:**
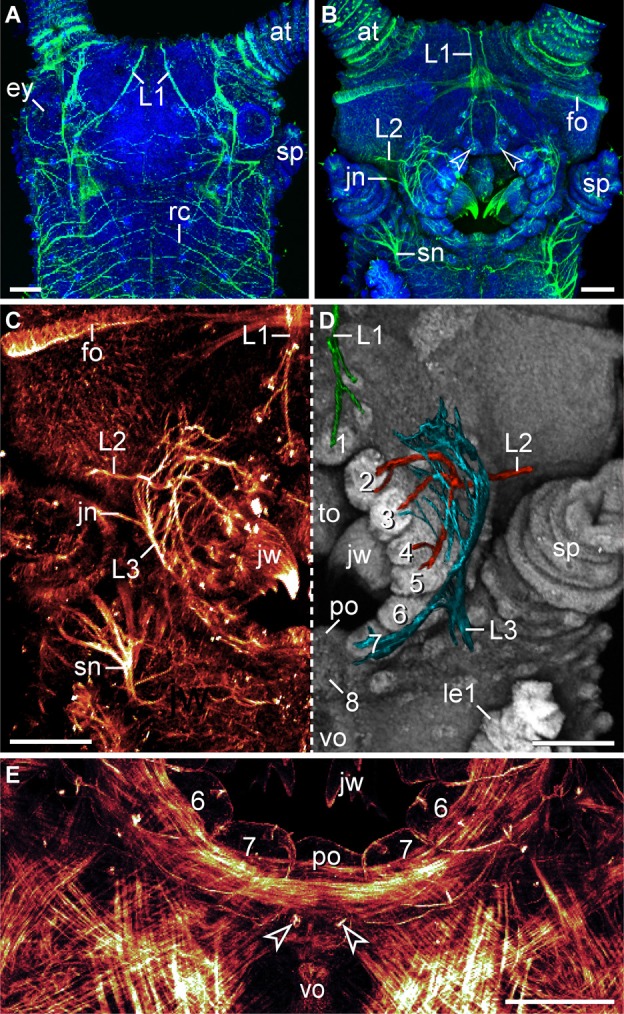
**Characteristics of nerves supplying the lip papillae in *Euperipatoides rowelli***. Maximum projection confocal micrographs of a late stage VII embryo. **(A,B)** Head in dorsal and ventral views, respectively. Double-labeling with an acetylated α-tubulin antibody (green) and the DNA marker Bisbenzimide (blue). Note the first pair of lip papillae nerves (L1) that originates dorsally and projects ventrally to supply the anterior-most lip papillae (arrowheads). **(C,D)** Details of the same embryo as in **(B)**. **(C)** Anti-acetylated α-tubulin immunolabeling (glow mode). Note the dense network of fibers formed by the second (L2) and third lip papillae nerves (L3). **(D)** The same portion of the embryo as in **(C)** but mirrored. The three lip papillae nerves (L1, L2, and L3, highlighted by artificial colors) were superimposed on the DNA-labeled surface of the head to illustrate their relationship to the lip papillae. **(E)** Ventral mouth portion of an embryo in ventral view to demonstrate the origin of the posterior most lip papilla (number 8) from a paired anlage (arrowheads). The lateral lip papillae surrounding the mouth opening are numbered. Note that the paired anlage occupies a position between the ventral and the preventral organs of the slime papilla segment. Phalloidin-rhodamine labeling. Abbreviations: at, antenna; ey, eye; fo, developing frontal organ; jn, jaw nerve; jw, jaw; L1–L3, lip papillae nerves 1–3; le1, first walking leg; po, preventral organ; rc, ring commissure; sn, slime papilla nerve; sp, slime papilla; to, tongue; vo, ventral organ. Scale bars: 100 μm **(A–E)**.

The anlagen of lip papillae surrounding the definitive mouth opening of *E. rowelli* arise relatively late during embryogenesis, as they occur after the anlagen of the anterior limbs and eyes have formed (Figures 4A–L). The second pair of lip papillae (number 2 in Figure 4L) appears first. It arises as an ectodermal fold anterior to the anlagen of jaws, behind the posterior border of the antennal segment (Figure 4C). During further development, this pair of lip papillae moves medially and is incorporated into the anterolateral wall of the definitive mouth opening (Figures 4D–L). The subsequent five pairs of lip papillae (numbered 3–7 in Figure 4L) appear rather simultaneously and, together with the second pair, they form a chain of bud-like structures, each with a single developing sensillum in the middle (Figure 4E). The number of sensilla on each lip papilla increases subsequently during development (Figures 4E–K). The second to seventh pairs of lip papillae contribute to the lateral walls of the definitive mouth opening (Figures 4E–L). The anterior-most, first pair of lip papillae develops next (number 1 in Figure 4L). Together with two pairs of additional, smaller papillae, it arises anteriorly in the antennal segment and then migrates ventrally to take up a position in the anterior wall of the definitive mouth (Figures 4F–L). The unpaired posterior lip papilla develops last (number 8 in Figure 4L). It originates from a pair of papillae, which occur posterior to the developing mouth (Figures 4J–L, 5E). The two initially separate papillae fuse medially, thus dividing the preventral and ventral organs of the slime papilla segment (Figure 4K). They give rise to the unpaired posterior lip papilla, which forms the posterior wall of the definitive mouth (Figure 4L). While the preventral organ is incorporated into the mouth cavity during development, the ventral organ persists as a roundish structure situated posterior to the unpaired, eighth lip papilla in post-embryonic stages (Figures 3A, 4L; see Oliveira et al., 2013 for further details on the development of the ventral and preventral organs).

### Innervation of the lip papillae in *Euperipatoides rowelli*

**Figure 6:**
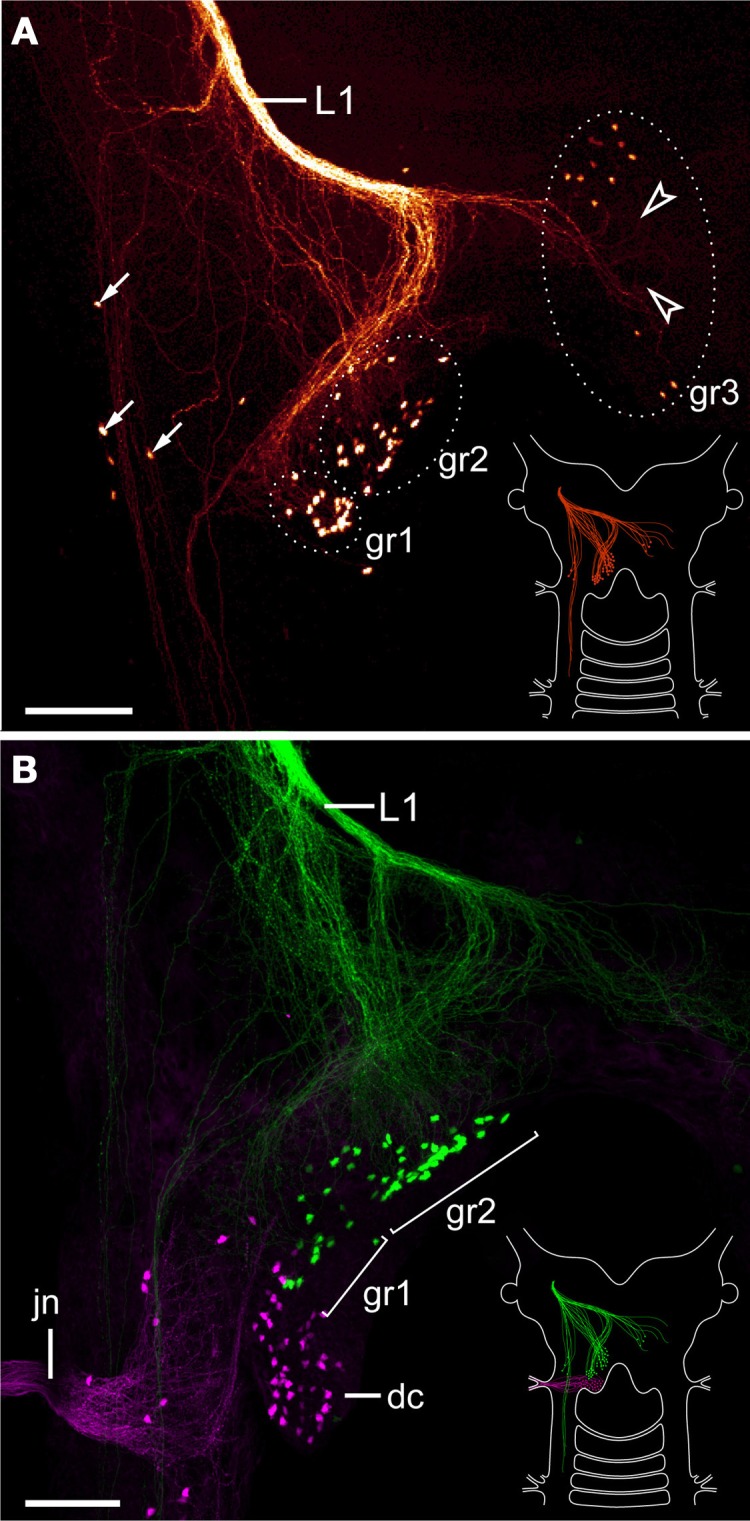
**Localization of neuronal somata associated with the first lip papillae nerve in *Euperipatoides rowelli***. Maximum projection confocal micrographs. Anterior is up in all images. **(A)** Retrograde fill of the first lip papillae nerve (L1) with dextran coupled to tetramethylrhodamine (glow mode). Note the position of neuronal somata in the posterior portion of the brain, a few additional lateral somata (arrows), and numerous fiber endings that are not associated with any somata (arrowheads). Diagram in the lower right corner illustrates the position of labeled somata and fibers in the brain. **(B)** Double-fill of the first lip papillae nerve with dextran coupled to fluorescein (green) and the jaw nerve with dextran coupled to tetramethylrhodamine (magenta) from the same body side to reveal the spatial relationship of the corresponding neuronal somata. Note that the somata associated with the first lip papillae nerve are located anterior to those innervating the jaw. Diagram in the lower right corner illustrates the position of labeled somata and fibers in the brain. Abbreviations: dc, deutocerebrum; gr1–gr3; first to third groups of neuronal somata associated with the first lip papillae nerve; jn, jaw nerve; L1, first lip papillae nerve. Scale bars: 100 μm **(A,B)**.

The lip papillae of *E. rowelli* are innervated by three pairs of nerves that are referred to as L1, L2, and L3 in the following, according to their antero-posterior arrangement within the head (Figures 5A–D; Supplementary Figure S2). The first nerve (L1) leaves the brain dorsally and projects antero-ventrally to supply the anterior-most lip papillae (Figures 5A,B). Retrograde fills of L1 with dextran coupled to a fluorochrome revealed that this nerve splits into several bundles and fibers with a stereotypic arrangement within the brain (Figures 6A,B). A major L1 bundle runs postero-medially and turns postero-laterally, where it terminates in a cluster of ~20 somata in the posterior portion of the brain (group 1 in Figure 6A). Only a few fibers from this bundle run further posteriorly into the nerve cord. An additional group of ~30 somata (group 2) is located antero-medially to this cluster, from which single fibers project anteriorly to join the major L1 bundle (Figures 6A,B). Another group of ~10 neuronal somata is situated in the contralateral brain hemisphere (group 3 in Figure 6A). These somata send off neurites that cross the midline to join the major L1 bundle.

In addition to these three groups of neuronal somata associated with L1, a few cell bodies are located laterally in the same hemisphere as L1 (arrows in Figure 6A). These cell bodies are associated with a lateral bundle of neurites, which continues posteriorly into the nerve cord (Figure 6A). In addition to fibers accompanied by neuronal somata, at least some neurites of L1 end blindly within the brain (arrowheads in Figure 6A). Double fills of L1 and the jaw nerve from the same brain hemisphere using dextran coupled to different fluorochromes revealed that all cell bodies associated with L1 are located anterior to the cluster of somata supplying the jaw (Figure 6B).

**Figure 7:**
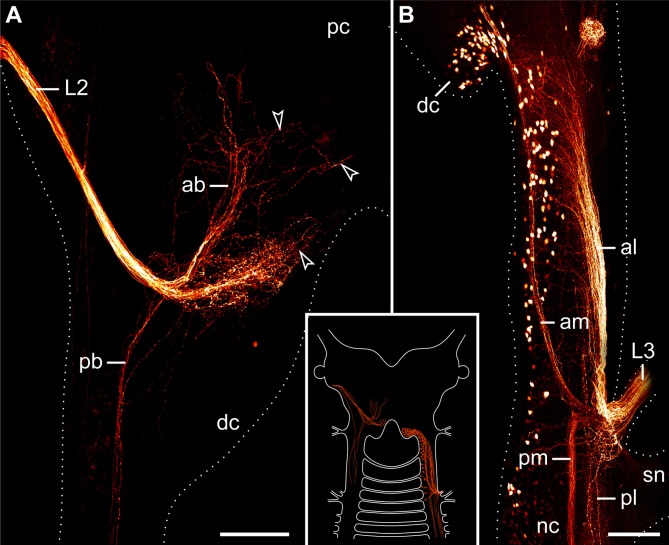
**Localization of neuronal somata associated with the second and third lip papillae nerves in *Euperipatoides rowelli***. Retrograde fills with dextran coupled to a fluorescent marker. Maximum projection confocal micrographs. Anterior is up in all images. Inset illustrates the position of the labeled somata and fibers in the central nervous system. **(A)** Fill of the second lip papillae nerve (L2). Note the lack of neuronal cell bodies associated with the L2 fibers (arrowheads). **(B)** Fill of the third lip papillae nerve (L3). Note the position of neuronal cell bodies in the deutocerebrum as well as further posteriorly outside the brain. Abbreviations: ab, anterior bundle; dc, deutocerebrum; al, anterior lateral bundle; am, anterior median bundle; L2 and L3, second and third lip papillae nerves; nc, nerve cord; pb, posterior bundle; pc, protocerebrum; pl, posterior lateral bundle; pm, posterior median bundle; sn, slime papilla nerve. Scale bars: 100 μm **(A,B)**.

**Figure 8:**
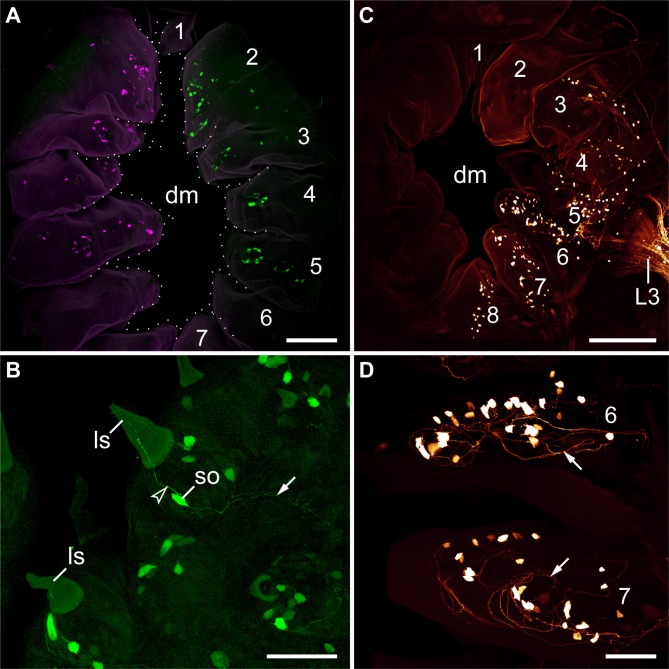
**Localization of somata of peripheral neurons associated with lip papillae in *Euperipatoides rowelli***. Anterograde fills with dextran coupled to the fluorescent markers tetramethylrhodamine or fluorescein. Maximum projection confocal micrographs. Lip papillae surrounding the mouth opening are numbered. **(A)** Overview (external perspective) showing the position of neuronal cell bodies within the lip papillae associated with the second pair of lip papillae nerves (L2). The nerves of each body side were labeled using two different fluorochromes (shown in magenta and green). Note that each nerve supplies the second to fifth pairs of lip papillae. The unpaired eighth papilla is not seen, as it lies beneath the seventh pair of lip papillae. **(B)** Details of lip papillae from the same preparation as in **(A)** (anterograde fill of L2; external perspective). Note the bipolar neurons associated with each sensillum (Storch and Ruhberg, 1977). Arrowhead points to a dendrite, arrow to an ascending axon. **(C)** Overview (internal perspective) showing the position of neuronal somata within the lip papillae associated with the third lip nerve (L3). Note that L3 receives fibers from neuronal cell bodies located in the second to eighth papillae. Corresponding somata within the second papilla are located further externally and, therefore, not seen in this micrograph (but see Supplementary Figure S3). **(D)** Details of lip papillae from the same preparation as in **(B)** (anterograde fill of L3; internal perspective). Note the high number of neuronal somata in the proximal portion of each lip papilla. Arrows point to fiber networks. Abbreviations: dm, definitive mouth opening; ls, lip sensillum; L3, third lip papillae nerve, so; soma of a bipolar neuron. Scale bars: 75 μm **(A,C)** and 50 μm **(B,D)**.

The second pair of lip papillae nerves (L2) originates from the ventro-lateral part of each brain hemisphere, after which each nerve splits into several branches that are associated with the second to fifth pairs of lip papillae (Figures 5B–D). Noticeably, the retrograde fills of L2 display no cell bodies within the brain. None of the L2 fibers crosses the midline and most of them terminate in a brush-like fashion in the posterior half of the brain (arrowheads in Figure 7A). Only a few fibers form an anterior bundle that ends blindly within the brain, whereas an additional posterior bundle extends into the nerve cord (Figure 7A). Our anterograde fills of L2 revealed ~150 somata of peripheral neurons within the second to fifth lip papillae on each side of the mouth (Figures 8A,B). Within each lip papilla, the neurons are arranged in groups of ~8 cells that lie at the bases of the lip sensilla. At least one cell in each group sends off two neurites in opposite directions: a short dendrite projecting into the lip sensillum and a long axon leading into the brain (Figure 8B).

The third pair of lip papillae nerves (L3) differs from L1 and L2 in that it does not arise from the brain but rather from the anterior region of the ventral nerve cords that innervates the slime papillae (Figure 7B). Imunocytochemical and neuronal tracing data show that L3 is associated with the lip papillae 2–8 (Figures 5C–E, 8C). Anterograde fills of L3 revealed a large number of ~200 somata of peripheral neurons, most of which are located near the inner surface of each lip papilla, although some of the somata do occur near the external surface of lip papillae (Figures 5E, 8C,D).

After entering the nerve cord, L3 separates into four major bundles of fibers: two anterior and two posterior bundles, respectively (Figure 7B). The two posterior bundles do not exhibit any cell bodies and might end blindly within the nerve cord further posteriorly, whereas the two anterior bundles are associated with neuronal cell bodies. The median bundle of the anterior L3 fibers extends into the brain, where it is associated with a cluster of ~100 somata that lie in the same region of the brain as neurons innervating the jaws (cf. Figures 6B, 7B). In contrast, the lateral bundle of the anterior L1 fibers is not associated with any somata located within the brain but rather with those distributed in the connecting pieces that link the nerve cords to the brain (Figure 7B). Notably, the number of neuronal somata associated with the lateral bundle of the anterior L1 fibers is not distributed evenly but decreases posteriorly, so that only a few somata are located in the region of the nerve cord supplying the slime papilla. Moreover, all these somata are located medially rather than laterally (Figure 7B).

## Discussion

### Morphogenesis of the definitive mouth suggests that the oral lip papillae arise from three anterior-most body segments in *Euperipatoides rowelli*

Despite detailed studies of the onychophoran head and associated structures, the segmental identity of the oral lip papillae remains unknown (e.g., Moseley, 1874; Sedgwick, 1885; von Kennel, 1888; Evans, 1901; Manton and Heatley, 1937; Pflugfelder, 1968; Walker and Campiglia, 1988; Eriksson and Budd, 2000; Walker and Tait, 2004; Mayer and Koch, 2005; Mayer et al., 2010; Oliveira et al., 2013). Our data on the morphogenesis of the mouth in the onychophoran *E. rowelli* revealed that the oral lip papillae of this species arise from three head segments. The first pair (“frontal processes” *sensu* Walker and Tait, 2004) can be allocated to the first (antennal or protocerebral) body segment, as it originates in the frontal region of the head and then migrates ventrally to take up a position in the anterior wall of the definitive mouth. This finding corresponds well with the ventral migration of these lip papillae described from other onychophoran species (Walker and Tait, 2004).

In contrast to the first pair, the segmental origin of the remaining six paired and one unpaired lip papillae (no. 2–8) of *E. rowelli* is less clear. However, since all of them arise behind the posterior border of the antennal segment (=cephalic lobes) and anterior to the *engrailed* domain of the slime papilla segment (cf. Eriksson et al., 2009), they might belong either to the second (jaw), third (slime papilla), or both segments. The anterior-most pair of these papillae (no. 2) arises antero-laterally to the anlagen of the jaws and is then followed by a chain of additional four pairs (no. 3–7) that appear rather simultaneously. Notably, an expression study of *decapentaplegic* (*dpp*) in embryos of *E. rowelli* revealed that this gene is expressed at the bases of the lip papillae 2–7, whereas no expression is seen within or next to the lip papillae 1 and 8 (Treffkorn and Mayer, 2013). The corresponding expression pattern of *dpp* at the bases of lip papillae 2–7 as well as their close spatial relationship to the anlage of the jaw suggest that these lip papillae belong to the same, i.e., the second body segment.

Our data on mouth development in *E. rowelli* further show that the unpaired, posterior lip papilla (no. 8) might be the only one that originates from the third (slime papilla) segment. Notably, this papilla arises from two separate anlagen, which subsequently move medially and fuse along the midline between the preventral and ventral organs of the slime papilla segment (Oliveira et al., 2013). After their fusion, the paired nature of the eighth lip papilla is still evident by the bilaterally symmetric arrangement of sensilla on its surface. This finding as well as the occurrence of paired posterior lip papillae in other onychophoran species (Oliveira et al., 2012) suggest that they are an ancestral feature of Onychophora, while the unpaired condition is derived. The independent origin of the eighth lip papilla from the remaining lip papillae in *E. rowelli* and its position between the preventral and ventral organs of the slime papilla segment suggest that this papilla belongs to the third body segment. Taken together, these findings imply that the oral lip papillae of *E. rowelli* originate from three anterior-most body segments.

### Neuronal tracing reveals that the lip papillae are mainly innervated by the proto- and deutocerebrum in *Euperipatoides rowelli*

**Figure 9:**
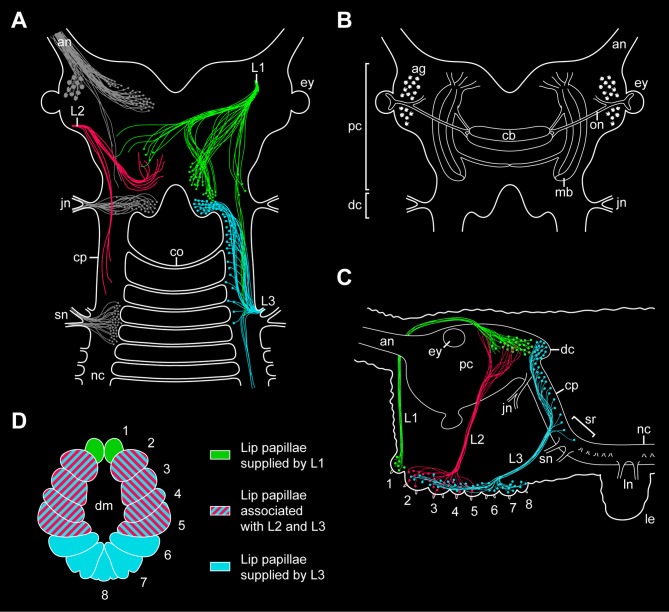
**Simplified diagrams summarizing the results of retrograde and anterograde fills of the lip papillae nerves in the onychophoran *Euperipatoides rowelli***. **(A)** Position of somata and fibers associated with the three lip papillae nerves, L1, L2, and L3, as revealed by retrograde fills. The innervation pattern of segmental head appendages (antenna, jaw, and slime papilla; see Mayer et al., 2010) is also shown (in gray) to demonstrate their spatial relationship to the lip papillae nerves. **(B)** Diagram of the onychophoran brain in dorsal view to demonstrate the position of major neural structures and neuropils (combined from various authors, e.g., Schürmann, 1987b; Strausfeld et al., 2006a). **(C)** Diagram of the onychophoran head in lateral view demonstrating the position of somata and fibers associated with the three nerves (L1, L2, and L3) supplying the lip papillae (numbered from 1 to 8). Based on series of Vibratome sections. **(D)** Color-coded diagram illustrating the innervation pattern of the lip papillae (numbered) by the corresponding nerves based on anterograde fills. Abbreviations: ag, antennal glomeruli; an, antennal nerve; cb, central body; co, first post-oral commissure; cp, connecting piece; dc, deutocerebral brain region; dm; definitive mouth opening; ey, eye; jn, jaw nerve; L1–L3, lip papillae nerves 1–3; le, first walking leg; ln, anterior and posterior nerves of the first walking leg; mb, lateral lobes of mushroom body; nc, nerve cord; on, optic nerve; pc, protocerebral brain region; sn, slime papilla nerve; sr, region of the nerve central nervous system supplying the slime papillae.

Although the trajectories of all three paired nerves associated with the onychophoran lip papillae have been described in detail, no information is available on the number of lip papillae supplied by each nerve (Fedorow, 1929; Hanström, 1935; Henry, 1948a; Eriksson and Budd, 2000—note that the authors use a varying and deviating nomenclature for these nerves). Our immunocytochemical and neuronal tracing data from *E. rowelli* revealed that the first pair of lip papillae nerves (L1) leaves the brain dorsally and then projects antero-ventrally to supply the anterior-most pair of lip papillae (Figures 9C,D). To some extent, this innervation pattern thus reflects the migration of the anterior-most lip papillae from a frontal to a ventral position during embryogenesis (Walker and Tait, 2004).

Our retrograde fills further show that the somata of descending fibers of L1 are located in the posterior half of the brain, posterior to the central body but still at the level of the mushroom bodies (Figures 9A–C). Since the segmental regions of the onychophoran brain do not show distinct physical borders (Schürmann, 1987b; Mayer et al., 2010), L1 cannot be assigned unambiguously to either the proto- or deutocerebrum. However, our double fills of L1 and the jaw nerve in *E. rowelli* show that the cell bodies associated with L1 are located anterior to the brain region innervating the jaw, i.e., the deutocerebrum. The position of L1 somata at the same level with the protocerebral neuropils (including the central body and the mushroom bodies) suggests that L1 belongs to the first rather than the second (=deutocerebral) segment. This assumption receives support from the embryonic origin of the anterior-most lip papillae associated with L1, which according to our developmental data clearly belong to the protocerebral segment.

While L1 contains both descending (motor) and ascending (sensory) fibers, our retrograde and anterograde fills of the second pair of lip papillae nerves (L2) in *E. rowelli* show that L2 exclusively consists of ascending fibers, as the corresponding neuronal cell bodies are located within the lip papillae and, therefore, outside the brain (Figures 9A,C). This suggests that L2 is not involved in the motor control of the lip papillae but rather has a sensory function. Although the afferent fiber endings of L2 seem to be located within the protocerebrum, the nerve itself is associated with the lip papillae 2–5 (Figures 9A,C,D), which according to our developmental data most likely belong to the second (deutocerebral) body segment. Due to this controversy regarding the innervation pattern and due to the lack of L2 somata within the brain, it is currently impossible to assign this nerve to either the proto- or deutocerebrum.

In contrast to L1 and L2, which directly connect to the brain, the third pair of lip papillae nerves (L3) is associated with the region of the central nervous system innervating the slime papillae (Fedorow, 1929; Hanström, 1935; Henry, 1948b; Eriksson and Budd, 2000; Mayer et al., 2010). However, only a few L3 somata are located in this region, whereas their number increases further anteriorly in the connecting pieces and about 40% of neuronal cell bodies are clearly located within the deutocerebrum (Figures 9A–C). This suggests that L3 is associated with the second and third body segments, which is in line with the innervation pattern of the lip papillae 2–8 by L3 (Figure 9D). While the lip papillae 2–7 arise from the second (jaw) segment during development, the eighth lip papilla originates from the third (slime papilla) segment. Since L3 is associated with two segments, this nerve might have evolved by a fusion of two ancestral nerves—one associated with the second and one with the third body segments. Accordingly, one would expect that the somata of neurons innervating the eighth lip papilla are located in the region of the nervous system supplying the slime papillae, whereas those innervating the lip papillae 2–7 should lie further anteriorly in the connecting pieces and in the deutocerebral region. To test this hypothesis, anterograde fills of single neurons associated with L3 will be necessary.

### Implications for the evolution of the brain in Ecdysozoa

In summary, while our developmental data show that the lip papillae of *E. rowelli* originate from three anterior-most body segments, retrograde and anterograde fills of the corresponding nerves display an intricate, overlapping innervation pattern. Among the three pairs of nerves, only L1 and L3 exhibit neuronal cell bodies within the brain, whereas L2 exclusively receives fibers from somata located in the lip papillae, making it difficult to assign this nerve to a particular brain region. Although most fibers of L2 seem to terminate in the protocerebrum, the nerve itself is associated with the oral lip papillae of the second body segment (Figures 9A–D). Another uncertainty concerns the innervation pattern of L3, which is mainly (albeit not exclusively) associated with the deutocerebrum but innervates the lip papillae of the second and third body segments. Therefore, L3 might have originated by a fusion of two pairs of ancestral nerves belonging to the second and third body segments.

Irrespective of this complexity in the innervation pattern of the onychophoran mouth, our data clearly show that the neuronal cell bodies supplying the lip papillae are not arranged in a ring-shaped pattern, as most neuronal somata of L1 and L3 are located dorsally within the brain (Figures 9A,C). Moreover, if a nerve ring were present, one would expect an association of either neuronal cell bodies or fibers innervating the mouth with the first post-oral commissure. However, our data clearly show that the nerve fibers innervating the lips do not pass to the contralateral side via the first or any other post-oral commissure. Instead, the only identified contra-lateral projections (associated with L1) are clearly located dorsally within the brain (Figure 9A). Hence, this innervation pattern is incompatible with the interpretation of the onychophoran brain “as a modified circumoral nerve ring, similar to that seen in the nematodes and other cycloneuralians” (Eriksson and Budd, 2000). Our findings instead support the assumption that the last common ancestor of Onychophora possessed a composite dorsal brain, which resembled the brain of extant onychophorans and arthropods (Schürmann, 1987a,b; Homberg, 1991; Strausfeld et al., 2006a; Mayer et al., 2010; Homberg et al., 2013).

Among the three panarthropod clades (Figure 1), tardigrades also show a typical dorsal brain, although there is some controversy regarding the number of segmental regions involved (Persson et al., 2012, 2013; Mayer et al., 2013a,b; Schulze et al., 2013). Hence, the last common ancestor of Panarthropoda most likely possessed a bilaterally symmetric, dorsal, ganglionic brain rather than a circumoral/circumpharyngeal, collar-shaped brain, which is a characteristic feature of cycloneuralians, including priapulids, loriciferans, kinorhynchs, nematodes and nematomorphs (Bullock and Horridge, 1965; Nielsen, 2012). Since we did not find any indication of such a circumoral ring in Onychophora, and since this feature does not occur in any other animal group, our findings support the hypothesis that the circumoral, collar-shaped brain is a synapomorphy of the cycloneuralian taxa (Ahlrichs, 1995; Nielsen, 2012). Thus, the last common ancestor of Ecdysozoa (Figure 1) most likely possessed a panarthropod-like, paired, ganglionic brain, which was modified in the cycloneuralian lineage.

## Author contributions

Christine Martin and Georg Mayer designed the experiments, carried out research and wrote the manuscript.

### Conflict of interest statement

The authors declare that the research was conducted in the absence of any commercial or financial relationships that could be construed as a potential conflict of interest.

## Supplementary material

Supplementary Figure S1 **Structure of the posterior-most lip papillae surrounding the mouth in the onychophoran *Euperipatoides rowelli***. Stereomicrograph; anterior is up. Arrowheads point to bilaterally arranged sensilla of the posterior-most lip papilla (number 8). Abbreviations: dm, definitive mouth opening. Scale bar: 100 μm.

Supplementary Figure S2 **Characteristics of nerves supplying the lip papillae in *Euperipatoides rowelli***. Maximum projection confocal micrographs of a late stage VII embryo. Lip papillae surrounding the mouth opening are numbered. The three lip papillae nerves (L1, L2, and L3) are highlighted by artificial colors. Abbreviations: dm, definitive mouth opening; jw, jaw; L1–L3, lip papillae nerves 1–3; po, preventral organ; sn, slime papilla nerve; to, tongue; vo, ventral organ. Scale bar: 100 μm.

Supplementary Figure S3 **Localization of somata of peripheral neurons associated with lip papillae in *Euperipatoides rowelli***. Anterograde fills of the third lip papillae nerve (L3) with dextran coupled to the fluorescent marker tetramethylrhodamine. Projections of confocal micrographs. Lip papillae surrounding the mouth opening are numbered. Overview of an external perspective (subset on the left) and an internal perspective (subset on the right) showing the position of neuronal somata within the lip papillae associated with the third lip papillae nerve (L3). Note that L3 receives fibers from neuronal cell bodies located in the second to eighth lip papillae. Abbreviations: dm, definitive mouth opening; L3, lip papillae nerve. Scale bar: 100 μm.
