# Supplementary figures and images for: Neuronal tracing of oral nerves in a velvet worm—Implications for the evolution of the ecdysozoan brain
Source: Front Neuroanat. 2014 Feb 26;8:7. doi: 10.3389/fnana.2014.00007 (PMC3935231; doi:10.3389/fnana.2014.00007)

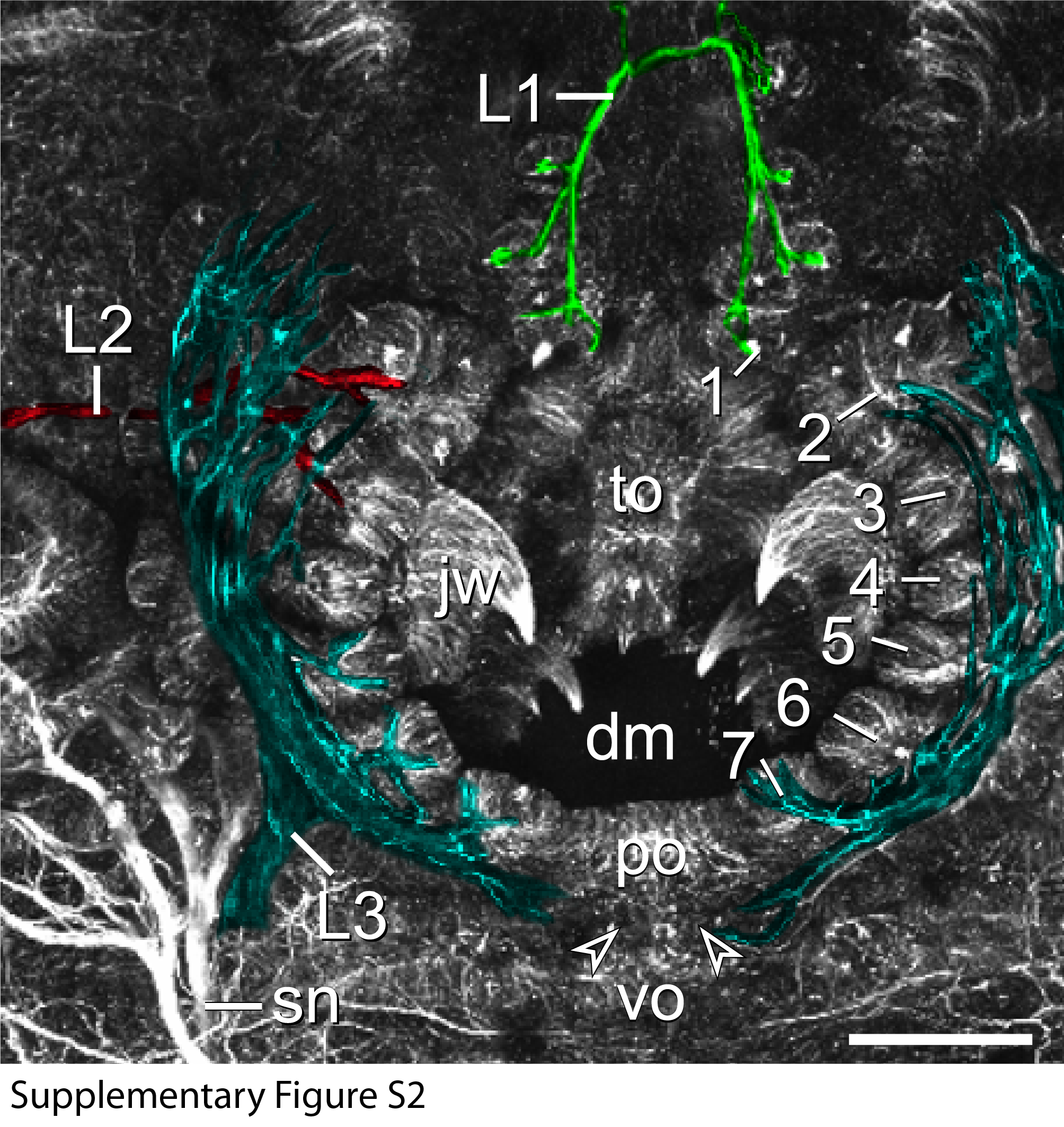

Supplement: Supplementary Figure S1 — Structure of the posterior-most lip papillae surrounding the mouth in the onychophoran Euperipatoides rowelli. Stereomicrograph; anterior is up. Arrowheads point to bilaterally arranged sensilla of the posterior-most lip papilla (number 8). Abbreviations: dm, definitive mouth opening. Scale bar: 100 μm. [file Presentation1.ZIP › 72197_Martin_Supplementary Figure_2.TIF]

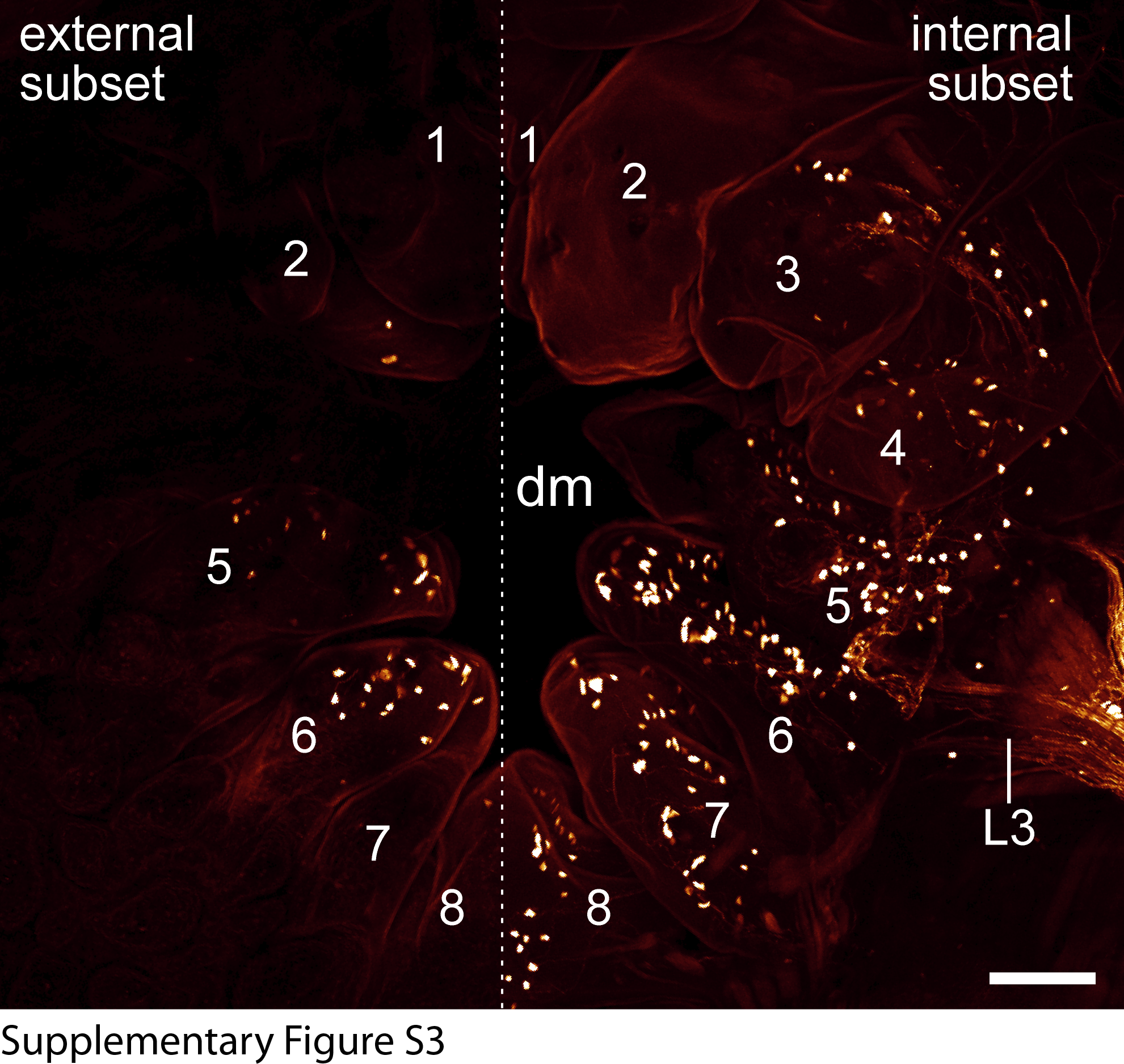

Supplement: Supplementary Figure S1 — Structure of the posterior-most lip papillae surrounding the mouth in the onychophoran Euperipatoides rowelli. Stereomicrograph; anterior is up. Arrowheads point to bilaterally arranged sensilla of the posterior-most lip papilla (number 8). Abbreviations: dm, definitive mouth opening. Scale bar: 100 μm. [file Presentation1.ZIP › 72197_Martin_Supplementary Figure_3.TIF]

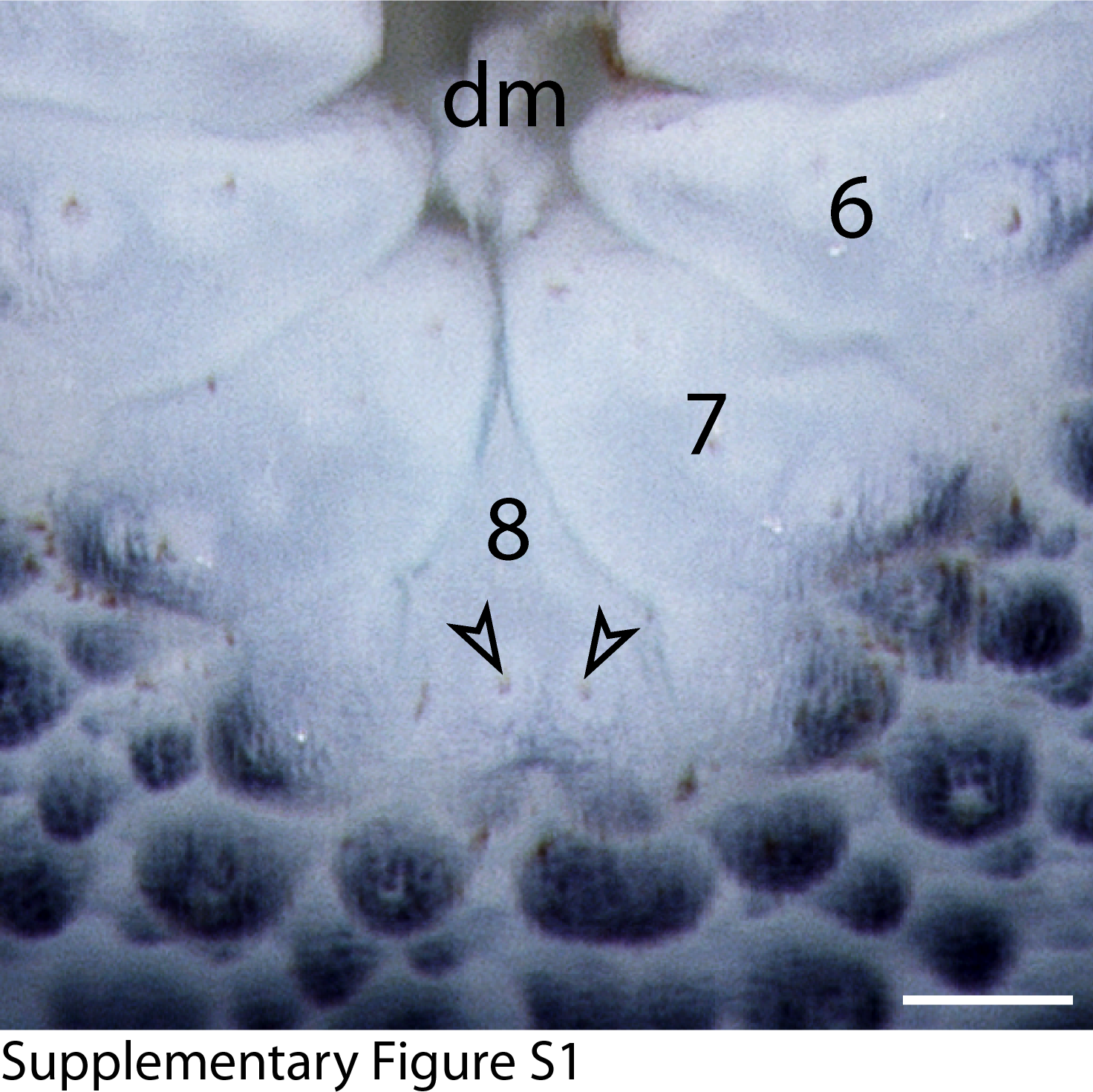

Supplement: Supplementary Figure S1 — Structure of the posterior-most lip papillae surrounding the mouth in the onychophoran Euperipatoides rowelli. Stereomicrograph; anterior is up. Arrowheads point to bilaterally arranged sensilla of the posterior-most lip papilla (number 8). Abbreviations: dm, definitive mouth opening. Scale bar: 100 μm. [file Presentation1.ZIP › 72197_Martin_Supplementary Figure_1.TIF]
